# Tracheopathia osteochondroplastica—A benign disorder with a daunting appearance

**DOI:** 10.1002/rcr2.1306

**Published:** 2024-02-20

**Authors:** Chia‐Hao Hu, Po‐Lan Su, Li‐Ting Huang, Chung‐Ta Lee, Tang‐Hsiu Huang

**Affiliations:** ^1^ Division of Chest Medicine, Department of Internal Medicine National Cheng Kung University Hospital, College of Medicine, National Cheng Kung University Tainan Taiwan; ^2^ Department of Diagnostic Radiology National Cheng Kung University Hospital, College of Medicine, National Cheng Kung University Tainan Taiwan; ^3^ Department of Pathology National Cheng Kung University Hospital, College of Medicine, National Cheng Kung University Tainan Taiwan

**Keywords:** airway, tracheal stenosis, tracheopathia osteochondroplastica

## Abstract

Tracheopathia osteochondroplastica (TO) is a rare and benign condition. It typically manifests as multiple osteocartilaginous nodules in the submucosa of the central airway. TO‐related clinical symptoms and physical signs are nonspecific. The bronchoscopic examination is helpful in establishing the diagnosis. Treatment for TO is mostly conservative and symptom‐oriented. The prognosis of TO is generally good, although cases of associated airway stenosis have been reported. In this case report, we describe the clinical, imaging, and histological features, and videoed bronchoscopic findings, of a middle‐aged male patient with incidentally diagnosed TO.

## Graphical abstract

Tracheopathia osteochondroplastica (TO) is a rare and benign condition of the central airway, but with a daunting appearance. In this case report, we describe the clinical, imaging, and histological features, and videoed bronchoscopic findings, of a middle‐aged male patient with incidentally diagnosed TO.

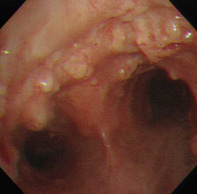

## INTRODUCTION

Tracheopathia (or tracheobronchopathia, if the bronchial walls are also involved) osteochondroplastica (TO) is an uncommon condition of the central airway of unclear aetiology that typically manifests as multiple nodular depositions of osteocartilaginous tissue in the tracheal (or tracheobronchial) submucosa.^1^, ^2^ Patients with TO can be asymptomatic or exhibit nonspecific respiratory symptoms. In this case report, we describe the clinical presentations, computed tomographic (CT) features, and videoed bronchoscopic findings of a patient with TO.

## CASE REPORT

**FIGURE 1:**
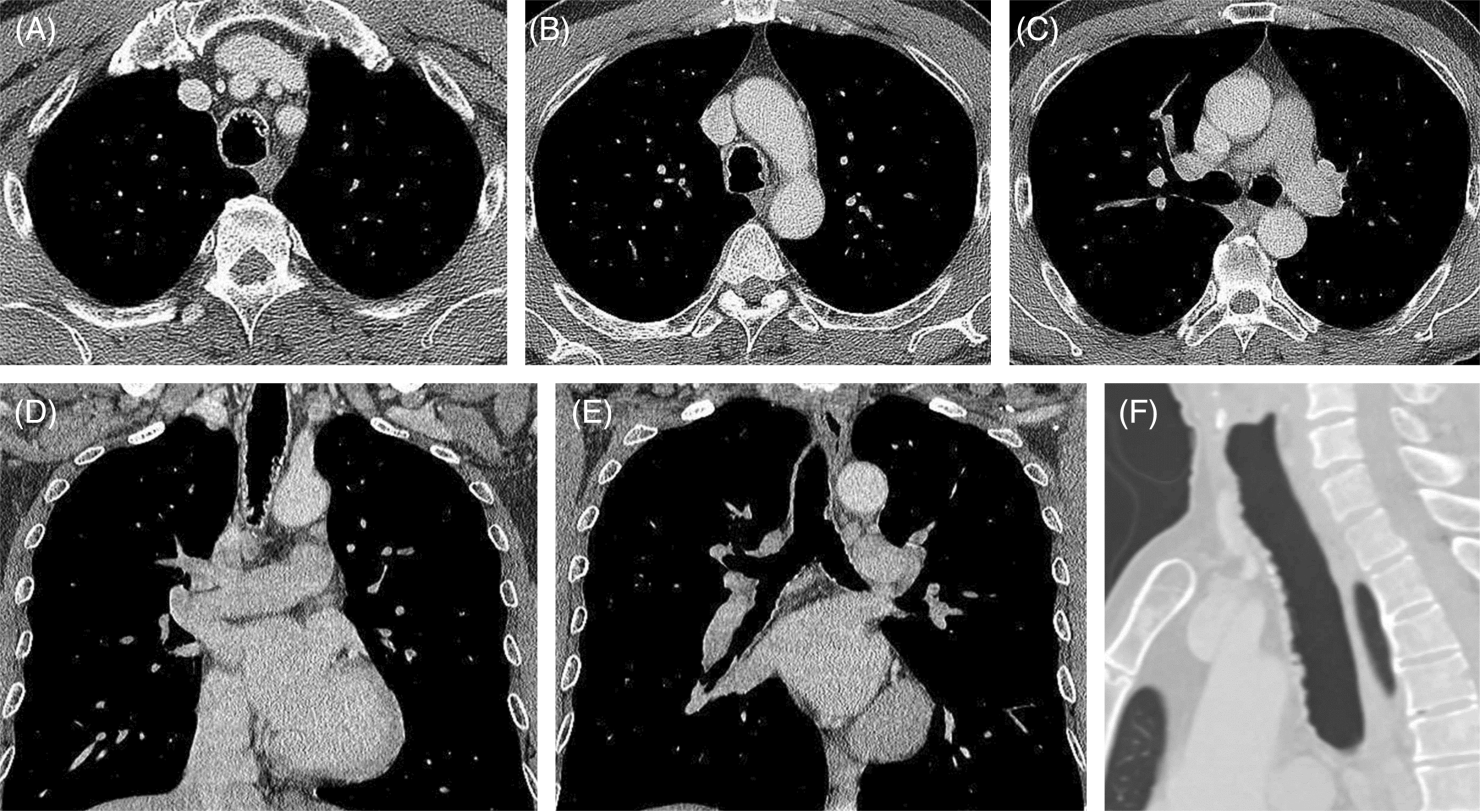
Thoracic CT images in transverse (A–C), coronal (D–E), and sagittal (F) views reveal multiple nodular ingrowths in the trachea, many of which exhibit mineral radiodensities. The posterior tracheal wall and the main bronchi are spared.

**FIGURE 2:**
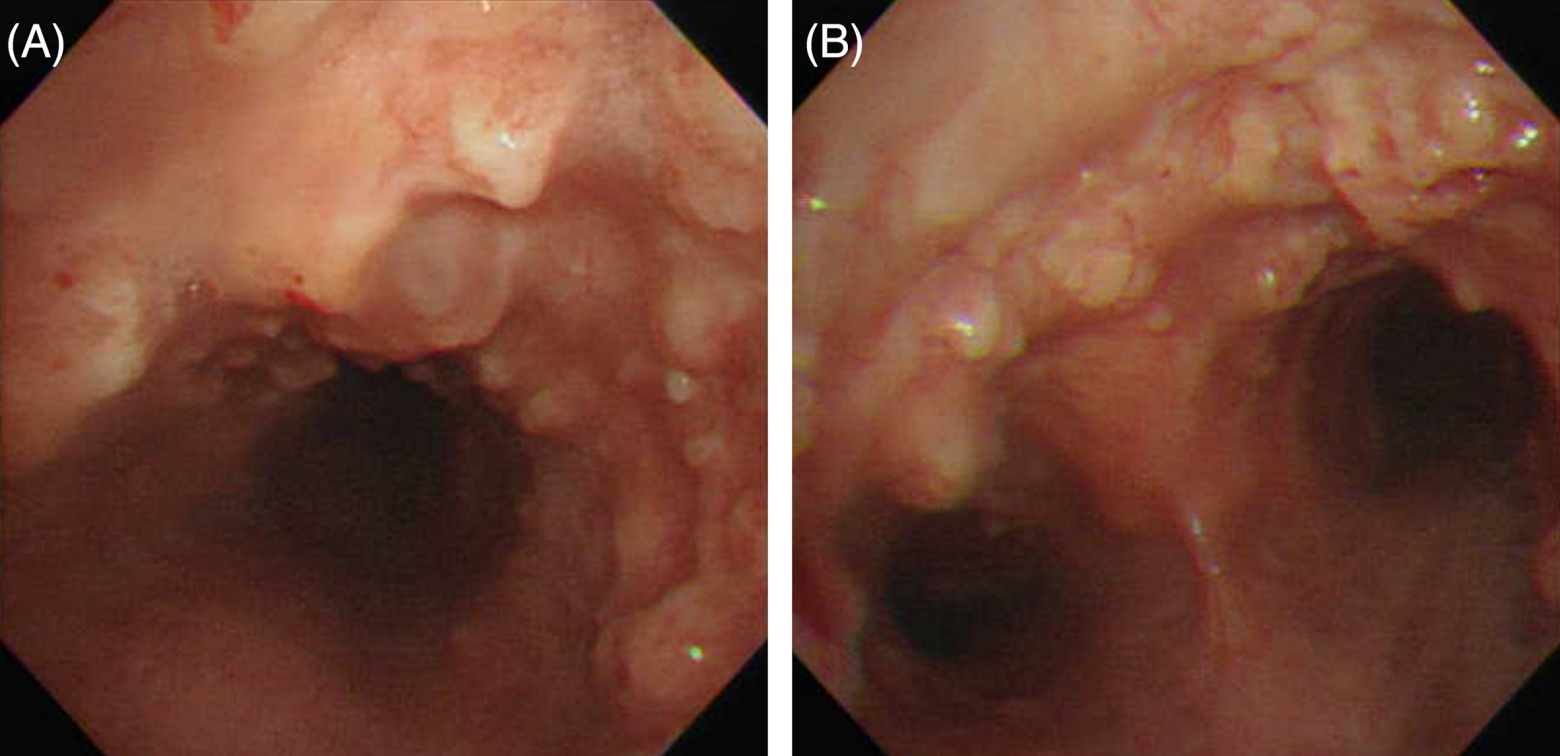
Numerous submucosal nodules arising from the antero‐lateral tracheal wall (but sparing the posterior wall) are observed under the white‐light bronchoscopy, starting from the subglottic zone (A) down to the carina (B).

**FIGURE 3:**
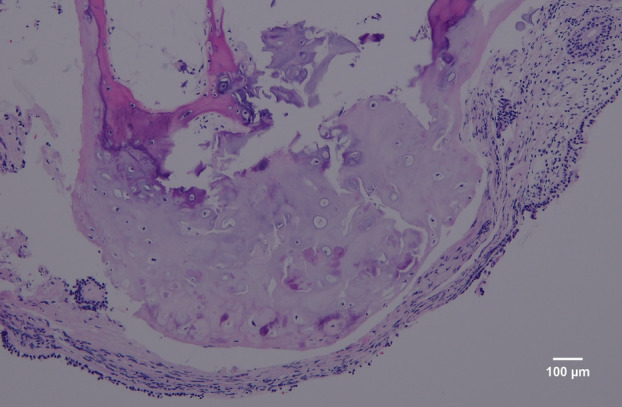
Microscopic examination of biopsied nodules reveals submucosal cartilaginous tissues with foci of ossification and overlying pseudostratified columnar epithelium (x 100; haematoxylin and eosin stain).

A 57‐year‐old non‐smoking man has long suffered from nonproductive cough. His past medical, family, and exposure histories were unremarkable. Following a mild airway infection 1 month earlier, the cough had aggravated, with associated exertional dyspnea. There was no associated fever, night sweating, chest pain, hemoptysis, or weight loss. He was transferred to our hospital for the bronchoscopic intervention of ‘endotracheal polypoid tumors’. The physical examinations including auscultation of the neck and chest were unrevealing. The thoracic CT showed multiple nodular ingrowths in his trachea, many of which exhibited mineral radiodensities (Figure 1). During white‐light bronchoscopy, numerous submucosal nodules arising from the antero‐lateral wall of the trachea were observed, starting from the subglottic zone to the carina. The posterior tracheal wall and the bronchial mucosa beyond the carina appeared normal (Video 1 and Figure 2). Histological examination of the biopsied nodules revealed submucosal cartilaginous tissues with foci of ossification and overlying pseudostratified columnar epithelium (Figure 3). There was no evidence of microbes, granuloma, amyloid deposition, or neoplasm. Results from relevant blood tests (including erythrocyte sedimentation rate, blood levels of calcium and phosphorus, the ratio of albumin versus globulin, antinuclear antibody, anti‐neutrophil cytoplasmic antibodies, and rheumatoid factor) were normal. Tracheopathia osteochondroplastica was diagnosed. The patient recovered well after symptom‐oriented treatments (which included oral tablets of dextromethorphan and fexofenadine). Follow‐up chest radiographs and pulmonary function tests 1 month and 18 months later revealed no radiographic evidence of tracheobronchial narrowing, and normal spirometry and lung volumes, respectively. He still has very mild and intermittent nonproductive cough but maintains an otherwise normal and active life.

**VIDEO 1:** This video was directly recorded during the white‐light bronchoscopy examination. As the scope gradually moves from the carina toward the subglottic zone, numerous submucosal nodules arising from the antero‐lateral tracheal wall are observed.

## DISCUSSION

TO is generally a benign entity despite its daunting appearance on radiographic images and under bronchoscopic examination.^1^, ^2^, ^3^ Symptoms and physical signs relating to TO can be either inconspicuous or, similar to those of our case patient, variable and nonspecific.^4^, ^5^ Bronchoscopy with or without tissue sampling is helpful to establish the diagnosis of TO, but like our case patient, many patients received bronchoscopic biopsy not just to rule in TO, but more importantly to rule out other important differential diagnoses such as tracheobronchial neoplasm, papillomatosis, mycobacterial infection, sarcoidosis, amyloidosis, granulomatosis with polyangiitis (formerly known as Wegner's granulomatosis), and relapsing polychondritis.^5^ Treatments for TO are mostly symptom‐oriented and may involve appropriate antimicrobial agents in case of associated airway infections.^2^, ^4^, ^5^ The overall long‐term prognosis of TO is favourable, tracking along a relatively stable course in most patients.^1^, ^2^, ^3^, ^4^, ^5^ However, cases of TO with progressive and severe airway stenosis have been reported, wherein advanced bronchoscopic ablative or stenting interventions, surgical resection or tracheoplasty, and tracheostomy may be considered.^2^, ^5^

In conclusion, we report the clinical, imaging, bronchoscopic, and histological features of a case of TO that was diagnosed incidentally due to exacerbating chronic cough after a mild airway infection. The gross appearance of TO may be alarming to clinicians and thereby summon biopsy and pertinent workups to exclude other malicious disorders. TO per se is a benign entity, but it is prudent to follow the patients for the potentially severe complication of progressive airway narrowing.

## AUTHOR CONTRIBUTIONS

CH Hu, TH Huang, and PL Su contributed to the concept, clinical data collection and review, and the drafting of the manuscript; LT Huang contributed to the collection and curation of radiographic images and the editing of the pertinent descriptions; CT Lee contributed to the collection and curation of histological images and the editing of pertinent descriptions; TH Huang contributed to the critical review of the manuscript and the correspondence.

## CONFLICT OF INTEREST STATEMENT

None declared.

## ETHICS STATEMENT

The authors declare that appropriate written informed consent was obtained for the publication of this manuscript and accompanying images.

## Data Availability

Data available on request due to privacy/ethical restrictions.
